# Impact of bridging thrombolysis on clinical outcome in stroke patients undergoing endovascular thrombectomy: a retrospective analysis of a regional stroke registry

**DOI:** 10.1007/s00234-020-02619-1

**Published:** 2020-12-16

**Authors:** Fatih Seker, Susanne Bonekamp, Susanne Rode, Sonja Hyrenbach, Martin Bendszus, Markus A. Möhlenbruch

**Affiliations:** 1 Department of Neuroradiology, Heidelberg University Hospital, Heidelberg, Germany; 2 Qualitätssicherung im Gesundheitswesen Baden-Württemberg GmbH, Stuttgart, Germany

**Keywords:** Stroke, Thrombectomy, Thrombolysis

## Abstract

**Purpose:**

It is unclear whether stroke patients undergoing endovascular thrombectomy (EVT) should receive bridging intravenous thrombolysis (IVT), if eligible. This study aims at analyzing the impact of bridging IVT on short-term clinical outcome.

**Methods:**

In a prospective regional stroke registry, all stroke patients with premorbid modified Rankin Scale (mRS) score of 0–2 who were admitted within 4.5 h after onset and treated with EVT were analyzed retrospectively. Patients receiving “IVT prior to EVT” (IVEVT) were compared to those undergoing “EVT only” regarding the ratio of good outcome, discharge mRS, mRS shift, hospital mortality, and occurrence of symptomatic intracranial hemorrhage.

**Results:**

In total, 2022 patients were included, 816 patients (40.4%) achieved good clinical outcome; 1293 patients (63.9%) received bridging IVT. There was no significant difference between both groups regarding the ratio of good outcome (IVEVT 41.4% vs. EVT 38.5%, *P* = 0.231), discharge mRS (median, IVEVT 3 vs. EVT 3, *P* = 0.178), mRS shift (median, IVEVT 3 vs. EVT 3, *P* = 0.960), and hospital mortality (IVEVT 19.3% vs. EVT 19.5%, *P* = 0.984). Bridging IVT was not a predictor of outcome (adjusted OR 1.00, 95% CI 0.79–1.26, *P* = 0.979). However, it was an independent predictor of symptomatic intracranial hemorrhage (adjusted OR 1.79, 95% CI 1.21–2.72, *P* = 0.005).

**Conclusions:**

The results of the present study suggest that bridging IVT does not seem to improve short-term clinical outcome of patients undergoing EVT. Nonetheless, there might be a subgroup of patients that benefits from IVT. This needs to be addressed in randomized controlled trials.

**Supplementary Information:**

The online version contains supplementary material available at 10.1007/s00234-020-02619-1.

## Introduction

Multiple randomized controlled trials have demonstrated that endovascular thrombectomy (EVT) is an effective treatment of acute ischemic stroke due to large vessel occlusion [1–8]. In some of these studies, additional treatment with intravenous thrombolysis (IVT) also known as bridging thrombolysis was a prerequisite for patient inclusion [2, 3, 6]. Nonetheless, EVT can be safely performed alone without prior IVT.

Numerous retrospective studies have been published comparing clinical outcome after EVT alone versus a combination of bridging IVT and EVT (IVEVT). Two recently published meta-analyses report that adding bridging IVT to EVT (IVEVT) does not result in better outcomes compared to EVT alone [9, 10]. Two other meta-analyses, however, reports better functional outcome in IVEVT patients [11, 12]. A post hoc analysis of the ASTER trial (Contact Aspiration Versus Stent Retriever for Successful Revascularization), for instance, reports that IVEVT is associated with lower 90-day mortality [13].

This raises the question whether bridging IVT is necessary or even beneficial in patients with large vessel occlusion undergoing EVT [14–16]. Currently, the randomized controlled trials SWIFT DIRECT, MR CLEAN NO-IV, and DIRECT-SAFE are recruiting patients. Interim analyses are not available, yet.

We, therefore, analyzed our regional real-world stroke registry with prospectively collected data of 2022 patients regarding short-term clinical outcome after EVT in patients with and without bridging thrombolysis (EVT alone vs. IVEVT). We hypothesize that outcome is similar in both groups.

## Methods

### Study design

A retrospective analysis of the prospectively documented stroke registry of Baden-Wuerttemberg was conducted. In Baden-Wuerttemberg, a state in southwest Germany with approximately 11 million inhabitants, all stroke centers are required to contribute data to this anonymized registry without the need of informed patient consent. The study is exempt from institutional review board approval.

This database is maintained by the Office of Quality Assurance in Hospitals in Stuttgart, Germany and is reported to include about 95% of all stroke patients in Baden-Wuerttemberg [17, 18]. The database has been maintained for quality assurance of IVT treatment. It includes demographic characteristics, time from onset to admission, NIHSS on admission, premorbid and discharge mRS score, and occurrence of symptomatic intracranial hemorrhage. Occlusion site, reperfusion success, and mRS at 90 days after stroke onset are not documented, though.

### Patient selection

Inclusion criteria for this retrospective study were (i) treatment with EVT between January 2014 and December 2017, (ii) premorbid mRS 0–2, and (iii) admission to hospital within 4.5 h after stroke onset. Patients with missing discharge information were excluded. The included patients were then divided into two groups: patients undergoing EVT alone and patients undergoing EVT and additionally receiving IV thrombolysis (IVEVT).

### Outcome measures

Primary outcome parameter was good clinical outcome defined as a discharge mRS of 0–2. Secondary outcome parameters were discharge mRS score, mRS shift (difference between premorbid mRS and mRS on discharge), hospital mortality, and occurrence of symptomatic intracranial hemorrhage (any intracranial hemorrhage associated with neurological deterioration).

### Statistical analysis

Statistical analysis was performed using R version 3.4.3 and RStudio version 1.1.383. Comparison between IVEVT and EVT was performed with two-tailed Student’s *t* test and Mann-Whitney *U* test for continuous data and Chi-square test for categorical variables. Univariate and multivariate regression analyses were performed for binary outcome analysis (good outcome, symptomatic intracranial hemorrhage, and hospital mortality). A *P* value of < 0.05 was considered statistically significant.

## Results

**Fig. 1:**
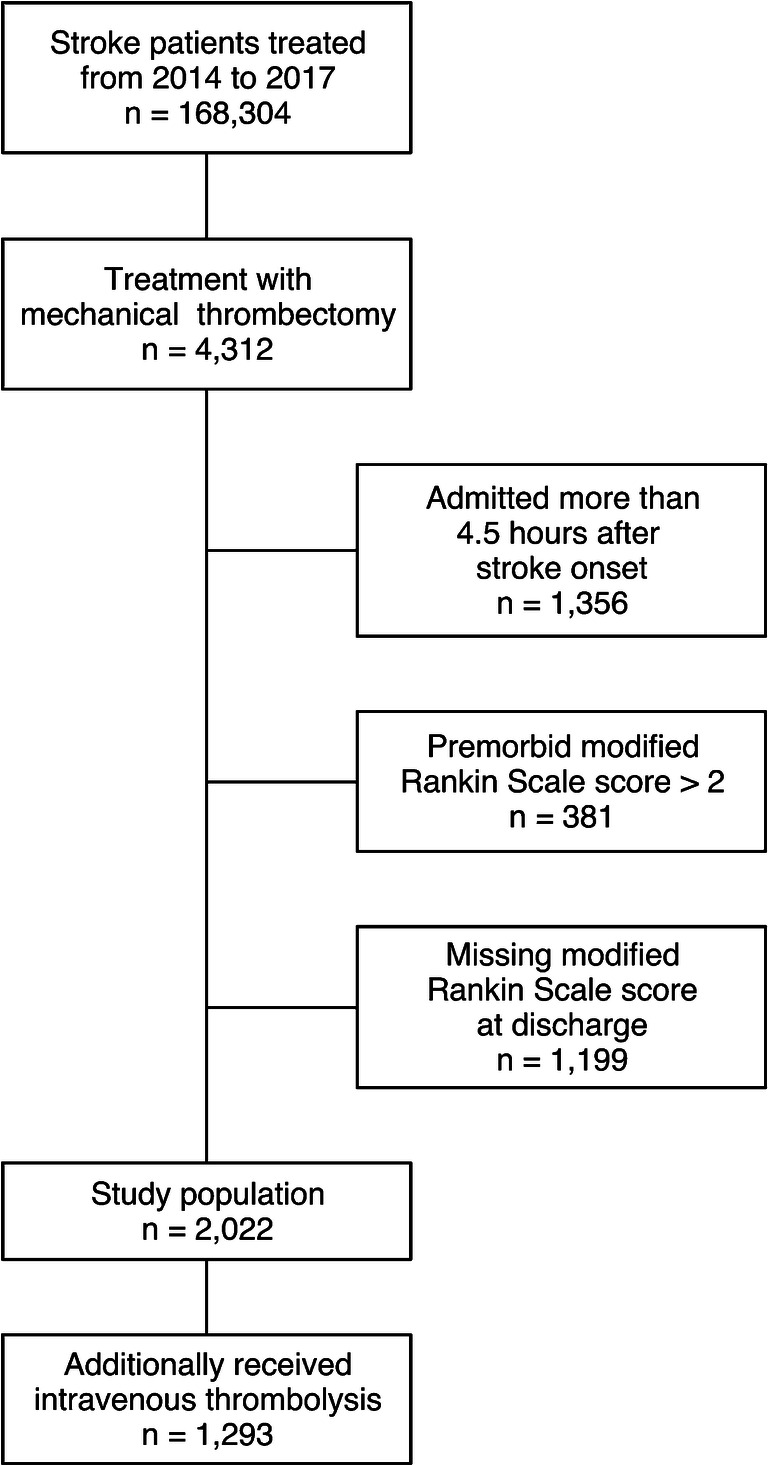
Flow chart of excluded and included patients

In total, 2022 patients were included in this study of which 816 patients (40.4%) achieved good clinical outcome;1293 patients (63.9%) received bridging IVT (Fig. 1). IVEVT patients were admitted slightly faster to a stroke center (median time, 110 vs. 120 min, *P* < 0.001). IVEVT patients were more frequently directly admitted to a comprehensive stroke center compared to patients undergoing EVT only (69.7% vs. 54.5%, *P* < 0.001) (Table 1).

**Table 1:** Patient characteristics

	Total (*n* = 2022)	IVEVT (*n* = 1293)	EVT (*n* = 729)	*P* value
Age (mean (SD))	72.0 (13.0)	71.4 (13.4)	73.2 (12.2)	0.002
Female (*n* (%))	958 (47.4)	634 (49)	324 (44.4)	0.053
Time from onset to admission (min, median (median, IQR))	120 (60–180)	110 (57–174)	120 (68–180)	< 0.001
Direct admission (*n* (%))	1298 (64.2)	901 (69.7)	397 (54.5)	< 0.001
Premorbid mRS (median (IQR))	0 (0–0)	0 (0–0)	0 (0–1)	< 0.001
Baseline NIHSS (median (IQR))	14 (8–19)	14 (9–19)	14 (8–19)	0.368
Comorbidities (*n* (%))				
Diabetes	406 (20.1)	244 (18.9)	162 (22.2)	0.080
Hypertension	1532 (75.8)	964 (74.6)	568 (77.9)	0.101
Atrial fibrillation	879 (43.5)	488 (37.7)	391 (53.6)	< 0.001
Previous stroke	280 (13.8)	155 (12.0)	125 (17.1)	0.002
Hypercholesterolemia	662 (32.7)	443 (34.3)	219 (30.0)	0.058

*EVT*, endovascular thrombectomy; *IQR*, interquartile range; *IVEVT*, bridging intravenous thrombolysis and endovascular thrombectomy; *mRS*, modified Rankin Scale; *NIHSS*, National Institutes of Health Stroke Scale; *SD*, standard deviation

**Fig. 2:**
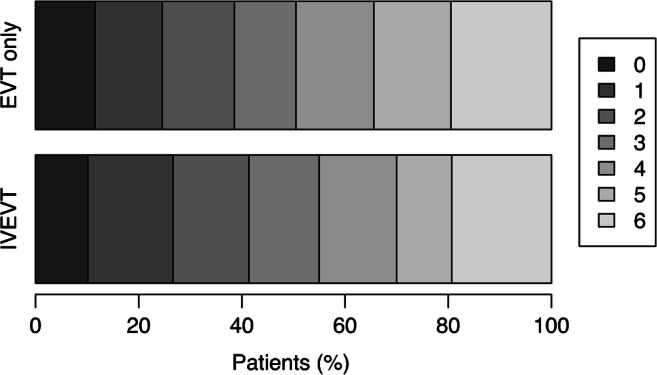
Modified Rankin Scale score at discharge of patients undergoing intravenous thrombolysis and endovascular thrombectomy (IVEVT) vs. patients undergoing thrombectomy only (EVT only). IVEVT patients did not have better clinical outcome compared to those undergoing thrombectomy only (*P* = 0.178)

The ratio of good outcome was similar in both groups (IVEVT 41.4% vs. EVT alone 38.5%, *P* = 0.231) (Table 2, Fig. 2). In univariate analysis, intravenous thrombolysis was not a predictor of good outcome (unadjusted OR 1.13, 95% CI 0.93–1.36, *P* = 0.213) (Table 3). Multivariate analysis showed that age, time from onset to admission, premorbid mRS, baseline NIHSS and diabetes were independent predictors of clinical outcome. However, intravenous thrombolysis was not an independent predictor (adjusted OR 1.00, 95% CI 0.79–1.26, *P* = 0.979) (Table 4).

**Table 2:** Clinical outcome

	Total (*n* = 2022)	IVEVT (*n* = 1293)	EVT (*n* = 729)	*P* value
Good outcome (*n* (%))	816 (40.4)	535 (41.4)	281 (38.5)	0.231
Hospital mortality (*n* (%))	392 (19.4)	250 (19.3)	142 (19.5)	0.984
Symptomatic intracranial hemorrhage (*n* (%))	158 (7.8)	120 (9.3)	38 (5.2)	0.001
Discharge mRS (median (IQR))	3 (1–5)	3 (1–5)	3 (2–5)	0.178
mRS shift (median (IQR))	3 (1–4)	3 (1–4)	3 (1–5)	0.960

*mRS*, modified Rankin Scale

**Table 3:** Univariate analysis of good outcome at discharge

	Unadjusted OR (95% CI)	*P* value
Age (per year)	0.96 (0.96–0.97)	< 0.001
Female	0.80 (0.67–0.96)	0.016
Time from onset to admission (per 10 min)	0.97 (0.95–0.98)	< 0.001
Direct admission	1.49 (1.23–1.80)	< 0.001
Premorbid mRS	0.42 (0.35–0.50)	< 0.001
Baseline NIHSS	0.88 (0.87–0.89)	< 0.001
Comorbidities		
Diabetes	0.59 (0.46–0.74)	< 0.001
Hypertension	0.72 (0.58–0.88)	0.001
Atrial fibrillation	0.56 (0.47–0.68)	< 0.001
Previous stroke	0.90 (0.69–1.17)	0.431
Hypercholesterolemia	1.08 (0.89–1.30)	0.449
Intravenous thrombolysis	1.13 (0.93–1.36)	0.213

*mRS*, modified Rankin Scale; *NIHSS*, National Institutes of Health Stroke Scale

**Table 4:** Multivariate analysis of good outcome at discharge

	Adjusted OR (95% CI)	*P* value
Age (per year)	0.97 (0.96–0.98)	< 0.001
Female	1.07 (0.86–1.34)	0.516
Time from onset to admission (per 10 min)	0.97 (0.95–0.99)	0.002
Direct admission	0.97 (0.73–1.28)	0.834
Premorbid mRS		
1 vs. 0	0.44 (0.31–0.60)	< 0.001
2 vs. 0	0.21 (0.12–0.33)	< 0.001
Baseline NIHSS	0.88 (0.86–0.89)	< 0.001
Comorbidities		
Diabetes	0.58 (0.43–0.76)	< 0.001
Hypertension	1.18 (0.90–1.55)	0.229
Atrial fibrillation	0.93 (0.74–1.18)	0.558
Previous stroke	1.18 (0.86–1.63)	0.308
Hypercholesterolemia	1.01 (0.80–1.28)	0.932
Intravenous thrombolysis	1.00 (0.79–1.26)	0.979

*mRS*, modified Rankin Scale; *NIHSS*, National Institutes of Health Stroke Scale

The ratio of hospital mortality was similar in both groups (IVEVT 19.3% vs. EVT alone 19.5%, *P* = 0.984). Multivariate analysis did not identify intravenous thrombolysis as an independent predictor of hospital mortality. However, the results suggest that there is tendency towards higher mortality (adjusted OR 1.30, 95% CI 0.98–1.74, *P* = 0.071) (Supplementary Tables I and II).

Symptomatic intracranial hemorrhage was significantly more frequent in IVEVT (9.3%) compared to EVT alone (5.2%) (*P* = 0.001). Multivariate analysis identified intravenous thrombolysis as an independent predictor of symptomatic intracranial hemorrhage (adjusted OR 1.79, 95% CI 1.21–2.72, *P* = 0.005) (Supplementary Tables III and IV). Good outcome was less frequent in patients receiving additional thrombolysis in case of symptomatic intracranial hemorrhage (16.7% vs. 43.9%, *P* < 0.001).

Discharge mRS (median, IVEVT 3 vs. EVT 3, *P* = 0.178) and mRS shift (median, IVEVT 3 vs. EVT 3, *P* = 0.960) were similar in both groups.

## Discussion

Historically, IVT was the first approved therapy of ischemic stroke and is still the standard of care in acute ischemic stroke. EVT has emerged as a more effective method in large vessel occlusions. The value of bridging IVT additional to EVT is an unresolved issue, though [14–16].

This retrospective analysis of a real-world regional stroke registry aims to provide more data on this issue. The stroke registry utilized in this study is mainly maintained for quality assurance of acute stroke treatment in Baden-Wuerttemberg. In total, 2022 patients were analyzed, a number which exceeds the study size of previous studies on the impact of bridging thrombolysis in EVT.

The main finding of this study is that patients receiving bridging IVT additional to EVT do not have an improved short-term clinical outcome compared to those undergoing EVT alone. The ratio of good clinical outcome and hospital mortality as well as median discharge mRS were similar in both groups. Furthermore, the ratio of bridging IVT was similar in patients with good and poor outcome.

Moreover, patients receiving bridging IVT showed a significantly higher ratio of symptomatic intracranial hemorrhage in this study. The type of hemorrhage was not documented in our regional stroke registry, though. While Kaesmacher et al. also reported an increased likelihood of hemorrhagic transformation in their meta-analysis, Fan et al. report similar ratios in EVT alone and IVEVT [9, 12].

In the literature, there are numerous studies on this topic with contradicting results. While two meta-analyses report that adding bridging IVT to EVT (IVEVT) does not result in better outcomes compared to EVT alone, two other meta-analysis reports better outcomes in IVEVT patients [9–12]. Probably, the inclusion criteria are the main reason for the contradicting results of these meta-analyses. For instance, while Phan et al. excluded studies with old generation devices, these were included in Mistry et al. [10, 11].

Post hoc analysis of randomized controlled trials such as ASTER, for instance, reports that IVEVT is associated with lower 90-day mortality [13]. Although these are randomized controlled trials, they are not controlled for IVT and need be interpreted cautiously.

The disparity of results may also point towards an unnoted aspect: There is probably a subgroup of stroke patients that is likely to benefit from IVT and a subgroup of patients that is likely to come to harm by IVT. Possible factors are thrombus density, thrombus histology, thrombus length, infarct core volume, etc. The studies that have been published up to now including the present studies are unable to provide these data. The potential strength of randomized controlled trials such as SWIFT DIRECT, MR CLEAN NO-IV, and DIRECT-SAFE is that they can identify factors that support or speak against bridging IVT.

Although our sample size exceeds those of prior studies on this topic, the study has several limitations. Of the 4312 patients undergoing EVT, 1199 patients (27.8%) had to be excluded due to missing discharge mRS which might lead to a bias in our study. These were mostly patients who were transferred to another hospital after thrombectomy. Nonetheless, our results are in accordance with prior studies [9, 10]. Furthermore, parameters such as occlusion site and recanalization success were not available. Patients with reperfusion after IVT only (i.e., without mechanical thrombectomy) are often underrepresented in thrombectomy studies, especially those patients who were transferred from another hospital after IVT and who then show recanalization on admission at the comprehensive stroke center. Unfortunately, the regional stroke database of the present study does not contain the number of patients with recanalization under IVT alone, either. Hence, the effect of IVT might be underestimated in this study. Moreover, no long-term follow-up but only discharge mRS was available to determine clinical outcome. An analysis of the NINDS-tPA study database has reported that early mRS at 1 week after stroke onset strongly correlates with 90-day mRS score and may be used as surrogate parameter [19].

## Conclusion

The results of the present study suggest that bridging IVT does not seem to improve short-term clinical outcome of patients undergoing EVT. Nonetheless, there might be a subgroup of stroke patients that still benefits from IVT. This needs to be addressed in randomized controlled trials.

## Supplementary information

ESM 1 (DOC 58 kb)

## Abbreviations

EVT: Endovascular thrombectomy
IVEVT: Intravenous thrombolysis with endovascular thrombectomy
IVT: Intravenous thrombolysis
mRS: modified Rankin Scale
NIHSS: National Institutes of Health Stroke Scale

## References

[1] Berkhemer OA, Fransen PSS, Beumer D, van den Berg LA, Lingsma HF, Yoo AJ, Schonewille WJ, Vos JA, Nederkoorn PJ, Wermer MJ, van Walderveen M, Staals J, Hofmeijer J, van Oostayen J, Lycklama à Nijeholt GJ, Boiten J, Brouwer PA, Emmer BJ, de Bruijn SF, van Dijk L, Kappelle LJ, Lo RH, van Dijk E, de Vries J, de Kort PL, van Rooij W, van den Berg J, van Hasselt B, Aerden LA, Dallinga RJ, Visser MC, Bot JC, Vroomen PC, Eshghi O, Schreuder TH, Heijboer RJ, Keizer K, Tielbeek AV, den Hertog H, Gerrits DG, van den Berg-Vos R, Karas GB, Steyerberg EW, Flach HZ, Marquering HA, Sprengers ME, Jenniskens SF, Beenen LF, van den Berg R, Koudstaal PJ, van Zwam W, Roos YB, van der Lugt A, van Oostenbrugge R, Majoie CB, Dippel DW, MR CLEAN Investigators (2015). A randomized trial of intraarterial treatment for acute ischemic stroke. N Engl J Med.

[2] Bracard S, Ducrocq X, Mas JL, Soudant M, Oppenheim C, Moulin T, Guillemin F, THRACE Investigators (2016). Mechanical thrombectomy after intravenous alteplase versus alteplase alone after stroke (THRACE): a randomised controlled trial. Lancet Neurol.

[3] Campbell BCV, Mitchell PJ, Kleinig TJ, Dewey HM, Churilov L, Yassi N, Yan B, Dowling RJ, Parsons MW, Oxley TJ, Wu TY, Brooks M, Simpson MA, Miteff F, Levi CR, Krause M, Harrington TJ, Faulder KC, Steinfort BS, Priglinger M, Ang T, Scroop R, Barber PA, McGuinness B, Wijeratne T, Phan TG, Chong W, Chandra RV, Bladin CF, Badve M, Rice H, de Villiers L, Ma H, Desmond PM, Donnan GA, Davis SM, EXTEND-IA Investigators (2015). Endovascular therapy for ischemic stroke with perfusion-imaging selection. N Engl J Med.

[4] Jovin TG, Chamorro A, Cobo E, de Miquel MA, Molina CA, Rovira A, San Román L, Serena J, Abilleira S, Ribó M, Millán M, Urra X, Cardona P, López-Cancio E, Tomasello A, Castaño C, Blasco J, Aja L, Dorado L, Quesada H, Rubiera M, Hernandez-Pérez M, Goyal M, Demchuk AM, von Kummer R, Gallofré M, Dávalos A, REVASCAT Trial Investigators (2015). Thrombectomy within 8 hours after symptom onset in ischemic stroke. N Engl J Med.

[5] Molina CA, Chamorro A, Rovira À, de Miquel A, Serena J, Roman LS, Jovin TG, Davalos A, Cobo E (2015). REVASCAT: a randomized trial of revascularization with SOLITAIRE FR® device vs. best medical therapy in the treatment of acute stroke due to anterior circulation large vessel occlusion presenting within eight-hours of symptom onset. Int J Stroke.

[6] Saver JL, Goyal M, Bonafe A, Diener H-C, Levy EI, Pereira VM, Albers GW, Cognard C, Cohen DJ, Hacke W, Jansen O, Jovin TG, Mattle HP, Nogueira RG, Siddiqui AH, Yavagal DR, Baxter BW, Devlin TG, Lopes DK, Reddy VK, du Mesnil de Rochemont R, Singer OC, Jahan R, SWIFT PRIME Investigators (2015). Stent-retriever thrombectomy after intravenous t-PA vs. t-PA alone in stroke. N Engl J Med.

[7] Albers GW, Marks MP, Kemp S, Christensen S, Tsai JP, Ortega-Gutierrez S, McTaggart R, Torbey MT, Kim-Tenser M, Leslie-Mazwi T, Sarraj A, Kasner SE, Ansari SA, Yeatts SD, Hamilton S, Mlynash M, Heit JJ, Zaharchuk G, Kim S, Carrozzella J, Palesch YY, Demchuk AM, Bammer R, Lavori PW, Broderick JP, Lansberg MG, DEFUSE 3 Investigators (2018). Thrombectomy for stroke at 6 to 16 hours with selection by perfusion imaging. N Engl J Med.

[8] Nogueira RG, Jadhav AP, Haussen DC, Bonafe A, Budzik RF, Bhuva P, Yavagal DR, Ribo M, Cognard C, Hanel RA, Sila CA, Hassan AE, Millan M, Levy EI, Mitchell P, Chen M, English JD, Shah QA, Silver FL, Pereira VM, Mehta BP, Baxter BW, Abraham MG, Cardona P, Veznedaroglu E, Hellinger FR, Feng L, Kirmani JF, Lopes DK, Jankowitz BT, Frankel MR, Costalat V, Vora NA, Yoo AJ, Malik AM, Furlan AJ, Rubiera M, Aghaebrahim A, Olivot JM, Tekle WG, Shields R, Graves T, Lewis RJ, Smith WS, Liebeskind DS, Saver JL, Jovin TG, DAWN Trial Investigators (2018). Thrombectomy 6 to 24 hours after stroke with a mismatch between deficit and infarct. N Engl J Med.

[9] Kaesmacher J, Mordasini P, Arnold M, López-Cancio E, Cerdá N, Boeckh-Behrens T, Kleine JF, Goyal M, Hill MD, Pereira VM, Saver JL, Gralla J, Fischer U (2019). Direct mechanical thrombectomy in tPA-ineligible and -eligible patients versus the bridging approach: a meta-analysis. J NeuroInterventional Surg.

[10] Phan K, Dmytriw AA, Lloyd D, Maingard JM, Kok HK, Chandra RV, Brooks M, Thijs V, Moore JM, Chiu AHY, Selim M, Goyal M, Pereira VM, Thomas AJ, Hirsch JA, Asadi H, Wang N (2019). Direct endovascular thrombectomy and bridging strategies for acute ischemic stroke: a network meta-analysis. J NeuroInterventional Surg.

[11] Mistry EA, Mistry Akshitkumar M, Obadah NM, Chitale Rohan V, James Robert F, Volpi John J (2017). Mechanical thrombectomy outcomes with and without intravenous thrombolysis in stroke patients. Stroke.

[12] Fan L, Zang L, Liu X, Wang J, Qiu J, Wang Y (2020) Outcomes of mechanical thrombectomy with pre-intravenous thrombolysis: a systematic review and meta-analysis. J Neurol [Internet]. [cited 2020 Mar 29]; Available from: 10.1007/s00415-020-09778-4 10.1007/s00415-020-09778-4 32140863

[13] Florent G, Bertrand L, Romain B, Jérôme B, Xavier B, Mikael M (2018). Mechanical Thrombectomy outcomes with or without intravenous thrombolysis. Stroke..

[14] Fischer U, Kaesmacher J, Pereira VM, Chapot R, Siddiqui AH, Froehler MT (2017). Direct mechanical thrombectomy versus combined intravenous and mechanical thrombectomy in large-artery anterior circulation stroke: a topical review. Stroke..

[15] Leslie-Mazwi TM, Chandra RV, Hirsch JA (2017). To tPA or not to tPA, that is the question. Am J Neuroradiol.

[16] Levy EI, Mokin M (2017). Stroke thrombolysis and thrombectomy—not stronger together?. Nat Rev Neurol.

[17] Gumbinger C, Reuter B, Wiethölter H, Bruder I, Rode S, Drewitz E, Habscheid W, Daffertshofer M, Diehm C, Neumaier S, Kern R, Ringleb PA, Hacke W, Hennerici MG (2013). A consecutive and prospective stroke database covers the state of Baden-Wuerttemberg with 10.8 million inhabitants in Germany. Neuroepidemiology..

[18] Seker F, Bonekamp S, Rode S, Hyrenbach S, Bendszus M, Möhlenbruch MA (2019) Direct admission vs. secondary transfer to a comprehensive stroke center for thrombectomy. Clin Neuroradiol [Internet]. [cited 2020 Jan 28]; Available from: 10.1007/s00062-019-00842-9 10.1007/s00062-019-00842-9 31605147

[19] Ovbiagele B, Saver JL (2010). Day-90 acute ischemic stroke outcomes can be derived from early functional activity level. Cerebrovasc Dis.

