# Supplementary material for: Impact of bridging thrombolysis on clinical outcome in stroke patients undergoing endovascular thrombectomy: a retrospective analysis of a regional stroke registry
Source: Neuroradiology. 2020 Dec 16;63(6):935–41. doi: 10.1007/s00234-020-02619-1 (PMC8128793; doi:10.1007/s00234-020-02619-1)
Supplement: Supplementary file 1 — (DOC 58 kb) [file 234_2020_2619_MOESM1_ESM.doc]

**Impact of bridging thrombolysis on clinical outcome in stroke patients undergoing endovascular thrombectomy:
A retrospective analysis of a regional stroke registry**

**SUPPLEMENTARY MATERIAL**

**Supplementary Table I. Univariate analysis for hospital mortality**

**Supplementary Table II. Multivariate analysis for hospital mortality**

**Supplementary Table III. Univariate analysis for symptomatic intracranial hemorrhage**

**Supplementary Table IV. Multivariate analysis for symptomatic intracranial hemorrhage**

**Supplementary Table I. Univariate analysis for hospital Mortality**

|  | Unadjusted OR (95% CI) | P Value |
| --- | --- | --- |
| Age (per year) | 1.06 (1.05 – 1.08) | < 0.001 |
| Female | 1.47 (1.18 – 1.83) | < 0.001 |
| Time from onset to admission (per 10 min) | 1.03 (1.02 – 1.05) | < 0.001 |
| Direct admission | 0.65 (0.52 – 0.81) | < 0.001 |
| Premorbid mRS | 1.79 (1.53 – 2.08) | < 0.001 |
| Baseline NIHSS | 1.10 (1.08 – 1.12) | < 0.001 |
| Comorbidities  Diabetes  Hypertension  Atrial fibrillation  Previous stroke  Hypercholesterolemia | 1.26 (0.96 – 1.64)  1.22 (0.94 – 1.60)  1.39 (1.11 – 1.73)  0.97 (0.69 – 1.32)  0.73 (0.57 – 0.93) | 0.085  0.149  0.004  0.835  0.011 |
| Intravenous thrombolysis | 0.99 (0.79 – 1.25) | 0.937 |

mRS, modified Rankin Scale; NIHSS, National Institutes of Health Stroke Scale.

**Supplementary Table II. Multivariate analysis for hospital mortality**

|  | Adjusted OR (95% CI) | P Value |
| --- | --- | --- |
| Age (per year) | 1.07 (1.05 – 1.08) | < 0.001 |
| Female | 1.04 (0.79 – 1.36) | 0.804 |
| Time from onset to admission (per 10 min) | 1.01 (0.99 – 1.04) | 0.252 |
| Direct admission | 0.70 (0.50 – 0.98) | 0.035 |
| Premorbid mRS  1 vs. 0  2 vs. 0 | 1.92 (1.36 – 2.70)  2.22 (1.48 – 3.32) | < 0.001  < 0.001 |
| Baseline NIHSS | 1.10 (1.08 – 1.12) | < 0.001 |
| Comorbidities  Diabetes  Hypertension  Atrial fibrillation  Previous stroke  Hypercholesterolemia | 1.27 (0.93 – 1.74)  0.69 (0.49 – 0.97)  0.75 (0.57 – 0.99)  0.73 (0.49 – 1.08)  0.84 (0.62 – 1.12) | 0.133  0.030  0.045  0.120  0.231 |
| Intravenous thrombolysis | 1.30 (0.98 – 1.74) | 0.071 |

mRS, modified Rankin Scale; NIHSS, National Institutes of Health Stroke Scale.

**Supplementary Table III. Univariate analysis for symptomatic intracranial hemorrhage**

|  | Unadjusted OR (95% CI) | P Value |
| --- | --- | --- |
| Age (per year) | 1.00 (0.99 – 1.01) | 0.822 |
| Female | 1.12 (0.81 – 1.55) | 0.492 |
| Time from onset to admission (per 10 min) | 0.99 (0.97 – 1.02) | 0.690 |
| Direct admission | 1.11 (0.79 – 1.58) | 0.537 |
| Premorbid mRS | 1.21 (0.95 – 1.52) | 0.112 |
| Baseline NIHSS | 1.04 (1.02 – 1.06) | < 0.001 |
| Comorbidities  Diabetes  Hypertension  Atrial fibrillation  Previous stroke  Hypercholesterolemia | 1.15 (0.76 – 1.68)  1.34 (0.90 – 2.04)  0.83 (0.59 – 1.15)  1.01 (0.61 – 1.58)  1.21 (0.86 – 1.69) | 0.498  0.160  0.264  0.977  0.269 |
| Intravenous thrombolysis | 1.86 (1.29 – 2.74) | 0.001 |

mRS, modified Rankin Scale; NIHSS, National Institutes of Health Stroke Scale.

**Supplementary Table IV. Multivariate analysis for symptomatic intracranial hemorrhage**

|  | Adjusted OR (95% CI) | P Value |
| --- | --- | --- |
| Age (per year) | 0.99 (0.98 – 1.01) | 0.257 |
| Female | 1.09 (0.77 – 1.55) | 0.624 |
| Time from onset to admission (per 10 min) | 0.99 (0.96 – 1.02) | 0.619 |
| Direct admission | 0.99 (0.63 – 1.56) | 0.963 |
| Premorbid mRS  1 vs. 0  2 vs. 0 | 1.47 (0.92 – 2.31)  1.55 (0.85 – 2.69) | 0.099  0.132 |
| Baseline NIHSS | 1.04 (1.02 – 1.06) | < 0.001 |
| Comorbidities  Diabetes  Hypertension  Atrial fibrillation  Previous stroke  Hypercholesterolemia | 1.11 (0.72 – 1.67)  1.54 (0.96 – 2.54)  0.85 (0.58 – 1.25)  0.94 (0.55 – 1.52)  1.13 (0.78 – 1.63) | 0.619  0.082  0.415  0.804  0.500 |
| Intravenous thrombolysis | 1.79 (1.21 – 2.72) | 0.005 |

mRS, modified Rankin Scale; NIHSS, National Institutes of Health Stroke Scale.
